# Emerging Therapeutic Approaches to Engage the Androgen Receptor for the Treatment of Castration-Resistant Prostate Cancer

**DOI:** 10.3390/cancers17233755

**Published:** 2025-11-25

**Authors:** Isla Henry, Rebecca Foreman, Lakshana Balachandran, Ethan Mortimer, Mohammad Asim

**Affiliations:** Department of Clinical and Experimental Medicine, University of Surrey, Guildford GU2 7XH, UK

**Keywords:** prostate cancer (PCa), castration-resistant prostate cancer (CRPC), metastatic castration-resistant prostate cancer (mCRPC), androgen receptor (AR), N-terminal domain (NTD), DNA-binding domain (DBD), ligand-binding domain (LBD), full-length androgen receptor (AR-FL), androgen receptor splice variants (AR-Vs)

## Abstract

Castration-resistant prostate cancer (CRPC) remains a major clinical challenge, with disease progression frequently occurring despite the use of potent androgen receptor (AR)-targeted therapies. As AR signalling continues to drive tumour growth in this setting, new therapeutic strategies are being developed to disrupt the AR axis through both direct and indirect mechanisms. This review highlights a selection of promising agents in preclinical or clinical development that represent the next generation of therapies targeting AR signalling. Direct approaches include novel agents that degrade the AR or target domains beyond the conventional ligand-binding domain, aiming to overcome resistance to existing anti-androgens. Indirect strategies are designed to interfere with AR function by modulating AR-associated transcriptional co-regulators, chromatin accessibility, and other regulatory proteins, such as splicing factors, that are critical for sustaining AR-driven gene expression in prostate cancer. Together, these therapies form the basis of emerging strategies to more effectively suppress AR activity in CRPC. This review discusses AR-activating mechanisms, the mechanisms of action of these agents, their clinical development status, and their potential to reshape future treatment paradigms in CRPC.

## Simple Summary

Prostate cancer remains one of the most prevalent cancers in men, and while current hormone therapies aim to block androgen receptor activity, many patients eventually develop resistance, leading to advanced disease. This review explores emerging therapeutic strategies designed to overcome this resistance by targeting the androgen receptor through both direct and indirect mechanisms. Direct inhibitors block specific regions of the receptor to prevent its activation, whereas indirect inhibitors disrupt related signalling pathways that sustain its activity. By summarising these novel approaches, including compounds that degrade or silence the receptor and those that interfere with its supporting networks, this review aims to provide insight into how these treatments could improve outcomes for patients with advanced, treatment-resistant prostate cancer and guide future research directions.

## 1. Introduction

Prostate cancer (PCa) is the most commonly diagnosed cancer in men and the second leading cause of cancer-related death worldwide. In the United Kingdom alone, around 55,000 new cases are diagnosed annually, accounting for nearly 28% of cancer diagnoses in men, with approximately 12,000 deaths each year. The lifetime risk is estimated at 1 in 6 to 8 men, with incidence rates peaking between 75 and 79 years of age [1]. Globally, the incidence of PCa is projected to nearly double from 1.4 million new cases in 2020 to 2.9 million by 2040, reflecting both demographic trends and improvements in detection [2].

Management and treatment of PCa follows a defined pathway, guided by stage and risk. Localised, low-risk disease is often managed through active surveillance, while radical prostatectomy and radiotherapy remain the primary curative options [3, 4, 5]. For patients with high-risk localised or locally advanced disease, androgen deprivation therapy (ADT) is frequently employed, either as monotherapy or in combination with radiotherapy [6]. ADT can be achieved surgically via castration but is more commonly administered through medical castration using gonadotropin-releasing hormone (GnRH) agonists and antagonists [7]. By lowering circulating androgen levels, ADT suppresses androgen receptor (AR) activation and protumour transcriptional activity, underscoring the central role of AR signalling in PCa biology [6].

Although ADT is initially effective in controlling advanced disease, its benefit for the majority of patients is temporary. Progression typically occurs within two to three years, resulting in the emergence of castration-resistant prostate cancer (CRPC) [8]. This clinical state arises because ADT exerts selective pressure that favours the survival and proliferation of tumour cells capable of persisting under androgen-deprived conditions. CRPC is characterised by rising prostate-specific antigen (PSA) levels, radiographic progression, and/or clinical deterioration despite castrate testosterone levels [8]. Importantly, most patients with CRPC will eventually develop metastases, at which point the disease is classified as metastatic CRPC (mCRPC), a stage associated with very poor prognosis and a median survival of only two to three years. Despite castrate conditions, disease progression in mCRPC continues to be driven largely by reactivated AR signalling [9].

The AR plays a central role in the pathogenesis and progression of PCa. It is a ligand-dependent transcription factor belonging to the nuclear hormone receptor superfamily and is encoded by the NR3C4 gene, located on the X chromosome at Xq11-Xq12. This 90 kb gene spans eight exons and encodes a 110 kDa protein composed of 919 amino acids [10]. Structurally, the AR consists of three major functional domains: the N-terminal domain (NTD), DNA-binding domain (DBD), and ligand-binding domain (LBD), connected by a flexible hinge region that also contains a nuclear localisation signal (NLS) responsible for AR nuclear import [11, 12].

While the three-dimensional structures of the DBD and LBD have been resolved, the NTD remains structurally uncharacterised due to its intrinsically disordered nature. The NTD contains activation function-1 (AF-1), which is constitutively active and includes transcriptional activation units TAU-1 and TAU-5, both essential for AR-mediated transcription of target genes [12]. In contrast, activation function-2 (AF-2), located within the LBD, becomes active only upon ligand binding, such as to dihydrotestosterone (DHT) [12, 13].

The DBD is highly conserved and mediates specific DNA recognition and binding to androgen response elements (AREs) within target gene promoters and enhancers. This is facilitated by two cysteine-rich zinc finger motifs, the first containing the P-box for DNA recognition and the second containing the D-box, which enables AR dimerization. These interactions promote the cooperative activity of AF-1 and AF-2 in transcriptional regulation [13]. Furthermore, TAU-1 and TAU-5 participate in ligand-dependent interdomain (N/C) interactions between the NTD and LBD, stabilising the AR dimer complex and reducing ligand dissociation [10].

Ligand binding within the LBD induces a conformational rearrangement in which helix 12 (H12) folds over the ligand-binding pocket, forming the AF-2 surface that recruits transcriptional coactivators and initiates AR signalling [10, 11, 12, 13]. Given its central role in ligand-mediated activation along with its well-characterised structure, the LBD has historically been a primary focus for drug discovery in prostate cancer, as its inhibition effectively suppresses AR-driven transcription and tumour progression [10].

Multiple adaptive mechanisms account for the persistence of AR activity in CRPC. These include AR gene and enhancer amplification, as well as point mutations in the LBD that broaden ligand specificity or convert antagonists into agonists. Such alterations enable tumour cells to sustain AR-driven transcription despite androgen depletion, underpinning resistance to standard AR-targeted therapies [8, 9, 14, 15].

The development of potent second-generation androgen receptor pathway inhibitors (ARPIs), including enzalutamide, apalutamide, darolutamide, and the androgen biosynthesis inhibitor abiraterone acetate, has significantly improved survival in advanced disease [16, 17, 18]. However, resistance is inevitable. In particular, therapies directed against the LBD have promoted the emergence of AR splice variants (AR-Vs), which lack part or all of the LBD and thereby evade inhibition [8, 19]. Cross-resistance among ARPIs further limits sequential treatment options, creating a therapeutic ceiling that emphasises the need for new strategies.

To address these limitations, a growing number of investigational therapies are being developed. Some act directly on the AR, such as degraders or inhibitors targeting domains beyond the LBD, while others modulate AR activity indirectly through disruption of transcriptional co-regulators, chromatin architecture, or splicing machinery. This review will discuss these emerging therapeutic approaches (Table 1), examining their mechanisms of action, developmental progress, and potential to reshape the therapeutic landscape of CRPC [19].

**Table 1:** A table to summarise all inhibitors discussed in this review.

Drug	Mechanism of Action	AR Domain Targeted	Effective Mutation/ Variant Coverage	Trial Phase	PSA Response Rate	IC_50_ Values	Key Limitations
EPI Compounds	Bind to Tau-5, preventing interactions between the AF-1 region, CREB-binding protein, and RAP74 [20]	NTD [20]	AR-FL and AR-Vs [21]	Phase I/II (EPI-7386) [22]	88% of patients achieved a ≥50% decline in PSA when this drug was combined with enzalutamide (EPI-7386) [22]	EPI-7386 has IC_50_ of 421 nM in LNCaP cell line [23]	Poor pharmacokinetics for EPI-002 [24] and IC_50_ is too high for EPI-001 [25]
QW07	Prevents interactions between the AR and CREB-binding protein thus inhibiting AR transcriptional activity [26]	NTD [27, 28]	AR-FL and AR-Vs [26]	Pre-clinical [26]	N/A	IC_50_ of 7.54 µM in 22Rv1 [26]	Lack of clinical data [27]
VPC compounds (VPC-17160, VPC-17281 and VPC-14449)	Prevents AR from interacting with chromatin, thereby reducing AR transcriptional activity [29]	DBD [29]	L702H, W742C and W742L at high concentrations [29]	Assumed pre-clinical as no clinical trials have been reported as of yet	Significant decrease in PSA levels [30]	IC_50_ for VPC-17160 is 2 µM and for VPC-17281 the IC_50_ value is 6 µM in LNCaP cell lines [30]	VPC-17160 has a short half-life [30] VPC-17281 shown to target AR-null cells [30] VPC-14449 has low efficiency when targeting AR-Vs [29]
Pyrvinium pamoate	Non-competitive AR inhibitor [31]	DBD [31]	AR-FL and AR-Vs [31]	Pre-clinical [32]	N/A	IC_50_ of ~8–30 nM in CWR22Rv1, LNCaP, LNCaP-C4-2, and LAPC4 [31]	Off target effects as targets the DBD (highly conserved across multiple nuclear receptors) [31]
PROTACs (ARV-110 and ARV-766)	Degradation of AR via the ubiquitin–proteasome degradation pathway [33]	Mostly LBD [33]	T878, H875 and L702H [34]	Phase I/II [35]	≥50% PSA declines in 50% of participants [35]	IC_50_ not publicly available	Many PROTACs are not effective against AR-Vs [36]
TAS3681	AR-LBD antagonist [37]	LBD [37]	F877L/T878, H875Y/T878A and AR-V7 [37]	Phase I [38]	PSA declined over 50% from baseline in a subset of patients [38]	IC_50_ was 18 nM for LNCaP cell line [37]	Potential short half-life [37]
CC-94676	Heterobifunctional cereblon-mediated ligand-directed degrader [39]	LBD [39]	L702H and H875Y [40]	Phase I/ Phase III [41]	PSA reductions greater than 30% were observed in 34% of patients across all dose levels [42]	IC_50_ values not publicly available	Ineffective against AR-Vs and less effective in patients who have received chemotherapy [40]
RIPTACs (H001, H003 and HLD-0915)	Forms a ternary complex with AR and effector proteins (EP) thereby inhibiting EP function [43, 44]	Undisclosed	AR-FL and AR-Vs [44, 45]	Phase I/II [46]	H001 and H003 significantly reduced PSA levels [43] HLD-0915 showed reduced PSA levels [45]	IC_50_ not publicly available	Large molecular weight, strict design requirements [43]
Asc-J9	Promotes AR degradation [47]	N/A	AR-FL and AR-V7 [48]	Assumed pre-clinical as no clinical trials have been reported as of yet	Reduced PSA levels [49]	IC_50_ not publicly available	Short half-life, low oral bioavailability, and limited aqueous solubility [48, 50]
ZEN-3694	BETi, ultimately inhibiting AR transcriptional activation [51]	N/A	AR-FL and AR-V7 [52]	Phase II [51]	8% of patients achieved a ≥50% decrease in PSA levels from baseline [51]	IC_50_ not publicly available	Dose dependent toxicities [51]
Hairpin pyrrole–imidazole polyamides	Binds minor groove of DNA, thereby inhibiting AR transcriptional activity [53, 54]	N/A	AR-FL and AR-V7 [53, 54]	Assumed pre-clinical as no clinical trials have been reported as of yet	N/A	IC_50_ not publicly available	May have off-target effects due to also inhibiting GR activity [55]
Niclosamide/ PDMX1001	Multiple suggested: Suppresses the FOXM1-mediated DNA damage response, inhibition of AR-V7, mitochondrial uncoupler and inhibiting mTORC1, STAT3, and Wnt/β-catenin pathways [56, 57]	N/A	AR-V7 and AR-FL [57]	Phase II [57]	5/9 patients achieved ≥50% PSA reductions when combined with abiraterone/prednisone [58]	IC_50_ values for niclosamide/PDMX1001 not publicly available	Poor bioavailability if only niclosamide is administered [57]

## 2. Direct AR-Targeting Compounds

### 2.1. NTD Inhibitors

#### 2.1.1. EPI Compounds

**Figure 1:**
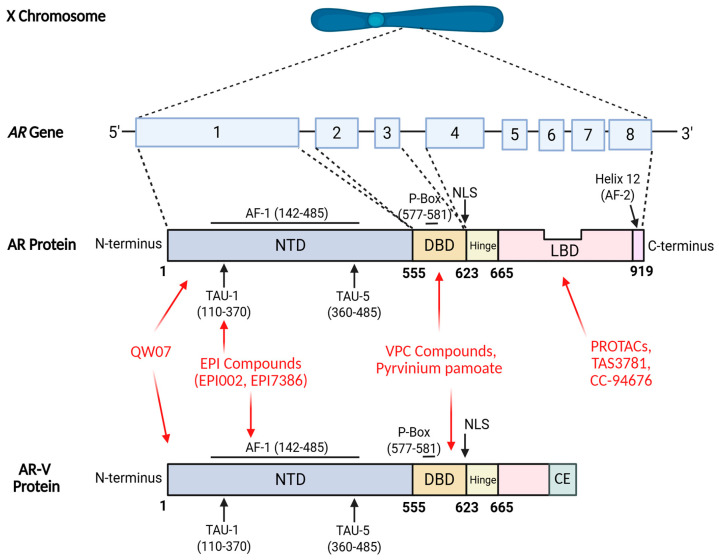
The structure of the AR and the binding sites of direct inhibitors. The AR gene is mapped to the long arm of the X chromosome at locus Xq11-Xq12. It comprises eight exons interspersed with introns of varying lengths and encodes the AR protein, a 919-amino-acid structure comprising three primary functional domains: the N-terminal domain (NTD), the DNA-binding domain (DBD), and the ligand-binding domain (LBD), along with a hinge region. Exon 1 encodes the NTD, exons 2 and 3 encode the DBD, while exons 4 through 8 encode the hinge region and LBD. Within the NTD, activation function 1 (AF-1) contains two activation units, TAU-1 and TAU-5. Activation function 2 (AF-2) is primarily located within the 12th helix of the LBD. The nuclear localisation signal (NLS) is located between the DBD and the hinge region. Amino acid residue numbers corresponding to these domains are indicated below the AR protein domain map [11, 12, 13]. QW07 targets and binds to the NTD of the AR. The EPI compounds target and bind to TAU-1 in the AF-1 region of the NTD [28, 29]. The VPC compounds and pyrvinium pamoate target and bind to the DBD; however, the VPC compounds bind more specifically to the P-box region within the DBD [29, 31]. The mentioned PROTACs, TAS3781 and CC-94676, target and bind to the LBD of the AR [33, 37, 39].

EPI compounds are direct AR inhibitors that bind to the NTD of the AR, which contains the AF-1 region. Within this region, the Tau-5 subdomain serves as a critical interface for protein–protein interactions essential for transcriptional activation. EPI compounds bind to Tau-5 (Figure 1), thereby preventing interactions between the AF-1 region, CREB-binding protein (CBP), and RAP74, and consequently inhibiting AR-mediated transcriptional activity [20]. Because EPI compounds act independently of the LBD, they can inhibit both full-length AR (AR-FL) and AR-Vs, and their binding is unaffected by androgen concentration [20].

In preclinical studies, EPI-001 significantly reduced the growth of LNCaP-95 cells, an androgen-independent and enzalutamide-resistant PCa line expressing both AR-FL and AR-V7, in vitro and in vivo [21]. The next-generation EPI compound, EPI-7386, was developed with approximately 20-fold greater potency than its predecessor EPI-002, enhanced metabolic stability in human hepatocytes, and the ability to induce tumour regression in multiple CRPC xenograft models [59]. EPI-7386 is currently undergoing Phase I/II clinical trials, both as monotherapy and in combination with enzalutamide. In an early combination study, 88% of patients achieved a ≥50% decline in PSA (PSA50), and 56% achieved PSA levels below 0.2 ng/mL [22]. In LNCaP cell lines, EPI-7386 has an IC_50_ of 421 nM [23].

However, there are a few limitations associated with the use of EPI compounds. The high IC_50_ value of EPI-001 is indicative of low potency, which then means greater doses of the drug would need to be taken by the patient [25]. Additionally, the poor pharmacokinetics of EPI-002 compounds means that even if the in vitro data look promising, when administered to patients the likelihood of the drug concentration in the blood being too low to have a significant effect on mCRPC is high [24, 60].

#### 2.1.2. QW07

QW07 is a tricyclic aromatic diterpenoid that functions as a direct AR inhibitor targeting the NTD (Figure 1). Information regarding the specific binding site within the NTD has not been disclosed [25, 26]. By binding to the NTD, QW07 inhibits AR transactivation and prevents interactions between the AR and CBP, thereby impairing AR binding to AREs. Targeting the NTD allows QW07 to inhibit the transcriptional activity of both full-length AR and AR-Vs [26].

Preclinical studies have demonstrated significant antitumor activity of QW07 in CRPC models [26]. In vivo experiments using castrated nude mice, designed to reflect CRPC conditions, showed that QW07 effectively inhibited tumour growth in 22Rν1 and VCaP cells resistant to enzalutamide [61]. At equivalent dosages, QW07’s efficacy was comparable to EPI-001, although QW07 exhibited a stronger binding affinity, with a lower dissociation constant (KD) of 1.4 µM compared to 2.0 µM for EPI-001 [26].

In vitro, QW07 inhibited the proliferation of LNCaP, C4-2, 22Rν1, and VCaP cells, with the latter three of these cell lines expressing AR-Vs [27]. Surface plasmon resonance assays indicated that QW07 displays a less stable and predictable binding association rate relative to enzalutamide, likely due to the disordered structure of the NTD [26]. Additionally, QW07 was more effective in AR-positive cancer cell lines than in AR-negative or healthy cells, as evidenced by lower IC_50_ values, suggesting selective cytotoxicity and a potentially improved therapeutic window. The IC_50_ of QW07 is 7.54 µM in 22Rv1 [26]. Currently, no clinical trials involving QW07 have been reported [26].

### 2.2. DBD Inhibitors

#### 2.2.1. VPC Compounds

VPC compounds represent a class of AR inhibitors that target the DBD (Figure 1) of the receptor [29]. One example, VPC-14449, binds to the DBD, specifically at the P-box, and prevents AR from interacting with chromatin, thereby reducing AR transcriptional activity. In reporter assays, VPC-14449 retained partial activity against AR-LBD point mutations; however, higher concentrations were required for less sensitive mutations such as L702H, W742C, and W742L [29].

Subsequent derivatives, including VPC-17160 and VPC-17281, which also inhibit at the AR-DBD P-box site, were developed based on VPC-17005, which effectively inhibited AR-positive cell growth but lacked sufficient metabolic stability for in vivo application. Both VPC-17160 and VPC-17281 were shown to significantly inhibit AR-V7 at 10 µM and AR-FL at 12 µM using reporter assays. They also caused significant reductions in secreted PSA levels, with respective IC_50_ values being 2 µM for VPC-17160 and 6 µM for VPC-17281 in LNCaP cell lines. These compounds also inhibited the proliferation of LNCaP cells and substantially reduced 22Rν1 cell line growth following VPC-17281 treatment [30]. These findings suggest that VPC compounds could be effective in treating CRPC. Whilst these compounds have not yet entered clinical trials, research suggests they are intended for future clinical testing for PCa.

Some key limitations of VPC compounds are the short half-life of VPC-17160. VPC-17281 has been shown to have some activity in AR-null cells, which is thought, by the inventor, to be a result of potential off-target effects; however, these effects have not been investigated further [30]. VPC-14449 has also shown a lower efficiency when targeting AR-Vs compared to wild-type AR [29].

#### 2.2.2. Pyrvinium Pamoate

Pyrvinium pamoate (PP), originally an anthelmintic, functions as a non-competitive AR inhibitor by binding the DBD (Figure 1) at the interface of the DBD dimer and the ARE minor groove. Targeting the DBD, the most conserved AR region, enables the inhibition of both full-length AR and AR-Vs. This may limit its usage due to it affecting other nuclear receptors, not just AR. PP prevents transcription by blocking RNA polymerase II binding at the transcription start sites [31].

In vivo studies in mice with 22Rν1 xenografts demonstrated delayed tumour growth compared to controls who were implanted with osmotic pumps containing vehicle (20% DMSO/80% PEG-400) without PP. However, no evidence of PP reducing tumour size in already established tumours has been shown. Through dual-energy X-ray absorptiometry, it was identified that PP has a cell-type specificity, demonstrated by its ability to have tissue-selective inhibition of AR activity in mice, as PP decreased prostate weight but did not affect lean body mass. However, it does have an adverse effect due to its ability to reduce bone mineral density. PP can also inhibit nuclear receptors such as GR and ER, but only in PCa cells, and has shown to be ineffective in non-prostate cells such as A549 and MCF7 [31]. The IC_50_ of PP is ~8–30 nM in CWR22Rv1, LNCaP, LNCaP-C4-2, and LAPC4 [31]. No clinical trials involving PP for prostate cancer have been reported as of yet [32].

### 2.3. LBD-Targeting AR Degraders

#### ARV-110 and ARV-766

**Figure 2:**
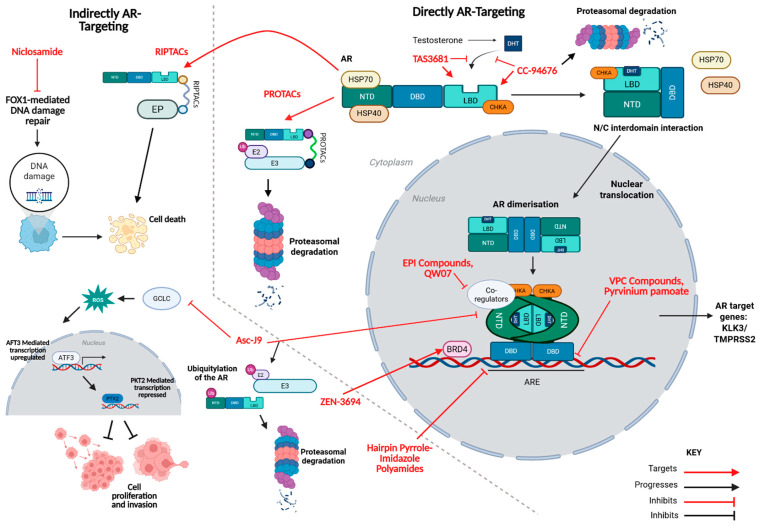
Mechanistic landscape of AR modulation: direct and indirect therapeutic strategies targeting AR signalling. The structure of the AR consists of three primary domains: the N-terminal domain (NTD), the DNA-binding domain (DBD), and the ligand-binding domain (LBD), accompanied by a small hinge region situated between the DBD and LBD (not depicted in the figure). In its inactive state, the AR resides in the cytoplasm, where it is bound to chaperone proteins, primarily heat shock proteins (HSPs) 70 and 40 [10]. Choline kinase alpha (CHKA) acts as a protein chaperone for the AR, thus stabilising it and preventing its degradation [62]. Upon ligand binding, such as dihydrotestosterone (DHT) (which is synthesised from testosterone) to the ligand-binding pocket of the LBD, the AR undergoes a conformational change. This change causes the dissociation of HSPs and facilitates the formation of an interdomain interaction between the NTD and the LBD, referred to as the N/C interdomain interaction. This interaction exposes the nuclear localisation signal, enabling the AR to translocate into the nucleus. Within the nucleus the AR dimerises and binds to androgen response elements (AREs) within canonical AR target genes (TGs), such as KLK3 and TMPRSS2 in order to regulate transcription. Subsequently, the N/C interdomain interaction is disrupted, allowing the AR to initiate transcription through interactions with coactivators [10, 11, 12]. Niclosamide exhibits anticancer potential through inhibition of the mTORC1, STAT3 and Wnt/β-catenin pathways. It may also act as a mitochondrial uncoupler and has been shown to suppress the FOXM1-mediated DNA damage response, indirectly increasing DNA damage and promoting apoptosis [57]. RIPTACs form a ternary complex with the AR and an effector protein (EP), thereby inhibiting the function of the EP, which is selective for cancer cells, ultimately leading to cell death [43, 44]. Asc-J9 interrupts the interaction between the AR and two of its coregulators, which results in an increase in inactive AR, which is then vulnerable to binding with the E3 ubiquitin ligase MDM2, leading to proteasomal degradation. Additionally, Asc-J9 inhibits the glutamate–cysteine ligase catalytic subunit (GCLC), reducing glutathione activity and increasing ROS levels. Elevated ROS upregulates activating transcription factor 3 (ATF3), which represses PTK2 transcription, inhibiting cell proliferation and invasion [47, 48, 63]. PROTACs facilitate the formation of a ternary complex upon simultaneous engagement of both the AR and the E3 ligase that brings the two proteins into close proximity, thereby activating the ubiquitin–proteasome degradation pathway. This results in polyubiquitination of the AR and subsequent degradation by the 26S proteasome. TAS3681 binds to the LBD of the AR, inhibiting androgens from binding and activating AR [35]. CC-94676 functions by degrading the AR but also antagonising the AR by competitively binding to the LBD [39]. EPI compounds bind to Tau-5, thereby preventing interactions between the AF-1 region and CREB-binding protein (CBP), which acts as a co-activator, consequently inhibiting AR-mediated transcriptional activity [20]. QWO7 inhibits AR by binding to the NTD, thereby inhibiting AR transactivation and prevents interactions between the AR and CBP, thereby impairing AR binding to AREs [26]. VPC compounds bind to the P-box of the DBD and prevent the AR from interacting with chromatin, thereby reducing AR transcriptional activity [29]. Pyrvinium pamoate functions as a non-competitive AR inhibitor by binding the DBD at the interface of the DBD dimer and the ARE minor groove. This prevents transcription by blocking RNA polymerase II binding at the transcription start sites [31]. ZEN-3694 binds to the acetyl-lysine recognition sites within bromodomains, such as BRD4, thereby competitively inhibiting their interaction with acetylated histones. This inhibition disrupts BET-mediated chromatin remodelling and consequently suppresses AR transcriptional activity [64]. Hairpin pyrrole–imidazole polyamides bind the minor groove of DNA, inducing conformational changes that affect protein–DNA interactions and modulates transcription. The illustrations shown in this figure are not to scale. The red arrows and inhibitors represent mode of action, whereas the black arrows and inhibitors represent the downstream effects.

Proteolysis-Targeting Chimeras (PROTACs) represent a promising therapeutic strategy for CRPC through their ability to induce targeted degradation of the AR. PROTACs are heterobifunctional molecules composed of two distinct ligands connected via a linker: one ligand binds the AR-LBD (Figure 1), whereas the other recruits an E3 ubiquitin ligase [33]. Commonly utilised E3 ligase-binding ligands are derived from cereblon, murine double minute 2 (MDM2), cellular inhibitor of apoptosis protein (cIAP), or the von Hippel-Lindau (VHL) tumour suppressor protein [34]. Upon simultaneous engagement of both the AR and the E3 ligase, PROTACs facilitate the formation of a ternary complex that brings the two proteins into close proximity, thereby activating the ubiquitin–proteasome degradation pathway. This results in polyubiquitination of the AR and subsequent degradation by the 26S proteasome (Figure 2) [33].

ARV-110 is an orally bioavailable cereblon-based PROTAC that demonstrated potent AR degradation, achieving greater than 95% reduction in AR protein levels in preclinical studies [65]. In Phase II clinical trials, ARV-110 produced ≥50% reductions in PSA levels in approximately 50% of patients harbouring T878X and H875Y mutations [33]. Although ARV-110 can degrade some clinically relevant AR mutations, its activity is limited against certain point mutations such as L702H and splice variants lacking the LBD. Therefore, this drug is not effective against AR variants such as AR-V7, which may limit its usage [36]. Optimisation of the AR-binding and E3 ligase-recruiting components of ARV-110 led to the development of ARV-766 (also known as luxdegalutamide or JSB462) [33]. ARV-766 exhibits enhanced efficacy against AR mutations, including T878, H875, and L702H, and is currently being evaluated in Phase I/II clinical trials [34]. In one study of 28 patients with LBD mutations (T878, H875, and L702H), ARV-766 induced ≥50% PSA declines in 50% of participants [35].

### 2.4. LBD Antagonists

#### TAS3681

TAS3681 is a small-molecule AR antagonist effective against both AR-FL and AR-V7. TAS3681 competes with androgens such as DHT for the LBD-binding pocket of the AR (the specific site has not been detailed publicly), inhibiting androgens from binding and activating AR. It is known that AR-FL heterodimerises with AR-Vs to drive transcription [66]. It is also known that AR-Vs require AR-FL to become active [67]. Moreover, PCa patients who become resistant to ADT and express AR-Vs always do so in conjunction with AR-FL [66]. All these observations suggest that in cell models or tumours which express both AR-FL and AR-Vs, by targeting the AR-FL, this may compromise AR-V function.

As a result of the antagonistic mechanism of TAS3681, this prevents AR dimerization and nuclear translocation, and as a result, the AR will not bind to the ARE on DNA. Therefore, there will be a reduction in AR transcriptional activity, which can result in the downregulation of both AR-FL and AR-V7 genes. Yoshida et al. found that TAS3681 reduced AR-FL and AR-V protein levels as well as the expression of AR-FL- and AR-V7-regulated genes. [37].

In addition, unlike enzalutamide, which loses efficacy against the F877L mutation due to partial agonist conversion, TAS3681 maintains antagonistic activity against multiple clinically relevant double mutations, including F877L/T878A and H875Y/T878A [37].

In preclinical studies using SAS MDV No. 3-14 cells, an enzalutamide-resistant derivative of LNCaP cells expressing AR-V7, TAS3681 dose-dependently reduced the expression of both AR-FL and AR-V7 in vitro and in vivo. These reductions were accompanied by decreased PSA levels and the suppression of AR-V7-mediated transcriptional activity [37]. TAS3681 also demonstrated enhanced antiproliferative activity compared with enzalutamide, with an IC_50_ of 18 nM versus 55 nM in LNCaP cells [37].

In early-phase clinical evaluation, once-daily administration of TAS3681 at 600 mg or 300 mg twice daily resulted in PSA declines of over 50% from baseline in a subset of patients. Additionally, 23.6% of patients receiving 600 mg once daily exhibited measurable tumour regression [38]. These results support TAS3681 as a potent agent capable of overcoming resistance mediated by AR mutations and splice variants. TAS3681 is currently in clinical trials, more specifically a Phase I study for patients with mCRPC [63].

### 2.5. LBD Antagonist and AR Degraders

#### CC-94676

CC-94676 is a first-in-class heterobifunctional cereblon-mediated ligand-directed degrader, which functions by degrading the AR but also antagonising the AR by competitively binding to the LBD (Figure 2) [39]. The exact binding site within the AR’s LBD has not been publicly disclosed. CC-94676 has demonstrated activity against wild-type AR and AR harbouring clinically relevant AR-LBD mutations such as L702H and H875Y in Phase I trials [40]. In an initial Phase I study, PSA reductions greater than 30% were observed in 34% of patients across all dose levels, and the treatment was generally well tolerated, with no Grade 4 treatment-related adverse events reported [42]. In a separate study, patients receiving 900 mg of CC-94676 achieved a median radiographic progression-free survival (rPFS) of 8.3 months, with chemotherapy-naïve patients demonstrating a longer median rPFS (16.5 months) compared to those with prior chemotherapy exposure (5.5 months) [40]. These findings suggest that CC-94676 may provide enhanced clinical benefit in chemotherapy-naïve patients. A new Phase III trial with CC-94676 began in March 2025 and is currently recruiting patients [41].

## 3. Indirect AR-Targeting Compounds

### 3.1. RIPTACs

#### 3.1.1. H001 and H003

Beyond direct AR inhibition, novel strategies such as regulated induced proximity targeting chimeras (RIPTACs) exploit AR as a means to selectively induce cancer cell death without directly antagonising receptor activity.

RIPTACs are heterobifunctional molecules comprising a ligand that binds to a target protein in cancer cells, such as the AR, and a second ligand that binds to essential effector proteins (EPs) present in all cells whilst both these ligands are connected via a linker (Figure 2) [43, 44]. The formation of a ternary complex between the AR and EP inhibits EP function selectively in cancer cells, leading to cell death [43]. As RIPTACs leverage the AR to induce cytotoxicity rather than directly inhibiting its transcriptional activity, they are thus considered indirect AR inhibitors in this review.

Two RIPTACs, H001 and H003, demonstrated greater efficacy compared to enzalutamide in vivo. In a castrated mouse model bearing VCaP xenografts, which are enzalutamide-insensitive due to AR-V7 expression, H001 and H003 significantly reduced PSA levels and promoted tumour regression [43]. However, enzalutamide co-administration impairs H001’s ability to form the ternary complex, as the AR-binding site for the RIPTAC is occupied [43]. Although information regarding the binding site for this RIPTAC is undisclosed, this may suggest that H001’s binding site is present within the AR-LBD.

#### 3.1.2. HLD-0915

HLD-0915 represents a first-in-class, oral RIPTAC therapeutic tested in humans and is currently undergoing Phase I/II clinical trials for mCRPC [46]. Following a recent announcement from the Phase I/II study evaluating HLD-0915, which uses BRD4 as the EP, HLD-0915 was said to exhibit clinical efficacy across diverse genomic contexts, including AR mutations, splice variants, and gene amplifications. In addition, HLD-0915 was well tolerated and demonstrated promising preliminary antitumour activity, evidenced by reductions in PSA levels and circulating tumour DNA [45]. This compound shows considerable potential as a next-generation therapeutic, offering a promising avenue for overcoming resistance mechanisms and improving outcomes in mCRPC.

### 3.2. AR Degradation via Coregulator Disruption

#### Asc-J9

Asc-J9 indirectly targets both full-length AR and AR-V7 [48]. Unlike enzalutamide, which merely inhibits AR-FL activity, Asc-J9 has been shown to promote AR degradation via coregulator disruption. In PCa cells, Asc-J9 interrupts binding between the AR and its coregulators, AR-associated protein 55 (ARA55) and AR-associated protein 70 (ARA70), which are enriched in stromal and luminal epithelial cells of the prostate [47]. This interruption ultimately leads to selective cell death (Figure 2). This selectivity was determined by a co-immunoprecipitation (Co-IP) assay and a mammalian two-hybrid assay. It is also strongly suggested that, by targeting these proteins, the AR is destabilised and made more vulnerable to binding with the E3 ubiquitin ligase MDM2 (Figure 2), as the AR is no longer able to perform its function [68].

Additionally, Asc-J9 inhibits the glutamate–cysteine ligase catalytic subunit (GCLC), reducing glutathione activity and increasing ROS levels. Elevated ROS upregulates activating transcription factor 3 (ATF3), which represses PTK2 transcription, inhibiting cell proliferation and invasion. Among the 105 genes upregulated by Asc-J9 but not by enzalutamide, 11 are linked to tumour suppression, indicating enhanced tumour suppressor synthesis [69].

However, in vivo studies were limited by subcutaneous tumour implantation, which may not accurately reflect orthotopic prostate conditions. Asc-J9 also exhibits short half-life, low oral bioavailability, and limited aqueous solubility, which constrains its clinical utility. Nanocarrier formulations have been proposed to overcome these limitations [48, 50]. In a positive light, Asc-J9 is shown to have few side effects and signs of toxicity in mice. There are no active clinical trials for Asc-J9 specifically targeting PCa; however, the extensive preclinical studies using Asc-J9 have supported the potential for future clinical trials in PCa [68].

### 3.3. Epigenetic Suppression with BET Inhibitors

#### ZEN-3694

ZEN-3694 is a pan-bromodomain extra-terminal (BET) inhibitor that indirectly suppresses AR-FL and AR-V7 activity by inhibiting transcriptional activation [51]. BET proteins function as epigenetic readers by binding to acetylated histones located in the promoter or enhancer regions of DNA. Through this interaction, they regulate gene expression by recruiting additional proteins that remodel chromatin structure. In doing so, BET proteins directly engage with the transcriptional machinery to either activate or repress gene activity [64, 70].

BET proteins comprise BRD2, BRD3, BRD4, and BRDT, which contain bromodomains that recognise acetylated lysine residues on histone tails. ZEN-3694 binds specifically to the acetyl-lysine recognition sites within these bromodomains, thereby competitively inhibiting their interaction with acetylated histones. This inhibition disrupts BET-mediated chromatin remodelling and consequently suppresses AR transcriptional activity [71]. ZEN-3694 is stated to successfully target the AR splice variant, AR-V7, in a preclinical characterisation prior to its entry into Phase I studies [52].

In a Phase Ib/IIa study, ZEN-3694 exhibited acceptable toxicity, with lower incidences of thrombocytopenia and gastrointestinal adverse effects relative to other BET inhibitors (BETis). The maximum tolerated dose was not reached in this study, and as such, the toxicity was deemed to be acceptable. In total, 8% of patients achieved a ≥50% decrease in PSA levels from baseline. Within the administered dose levels of 36 to 144 mg per day, 96 mg per day was determined to be the optimal dosage, as the effects of the drug cease to increase past that dosage. Nonetheless, dose reductions and discontinuations of ZEN-3694 administration were observed, highlighting dose-dependent toxicities. The percentage of patients needing dose reductions increased sharply between 96 mg and 120 mg of ZEN-3694, from 35% to 75%. However, when considering the number of patients given those doses, it was found that 31 patients were given 96 mg and only 4 patients were given 120 mg. The percentage of dose reductions or discontinuations at 120 mg is unreliable, as the sample size is too low and as a result, there is a greater chance of these dose reductions and discontinuations being due to another cause unrelated to ZEN-3694 dosages. Limitations of the study, including small sample size, non-randomised dose expansion, and few paired biopsies, restrict the reliable assessment of efficacy [51]. ZEN-3694 is currently being evaluated in Phase II clinical trials in comparison with enzalutamide [51].

### 3.4. AR Blockade Through DNA Binding Inhibition

#### Hairpin Pyrrole–Imidazole Polyamides

Hairpin pyrrole–imidazole polyamides have shown promise in indirectly inhibiting both the AR-FL and AR-V7 by blocking the AR-DNA interaction rather than the AR itself. These are synthetic oligomers that bind the minor groove of DNA, inducing conformational changes that affect protein–DNA interactions and modulates transcription [53, 54]. These oligomers are composed of N-methylpyrrole and N-methylimidazole units that form hairpin structures via γ-aminobutyric acid linkers and bind to the DNA via non-covalent bonds [55]. ARE-1 is a synthetic pyrrole–imidazole polyamide that binds to the ARE minor groove, reducing transcriptional activity of the AR and the glucocorticoid receptor (GR). This may be problematic as it could have off-target effects due to it also targeting and inhibiting the GR [55].

In vivo studies using LNCaP xenograft murine models demonstrated a 64% reduction in tumour growth after three treatment cycles compared with controls, which were LNCaP xenograft mice who received PBS vehicle. Additionally, tumour-bearing animals exhibited weight loss, whereas tumour-free animals did not [53]. This is important as it suggests it has low toxicity in healthy mice, but it does also suggest that it may be important to monitor the body weight if treatment is given to cancer patients. Hairpin pyrrole–imidazole polyamides have not yet advanced to clinical trials for PCa; however, pre-clinical research as discussed above has shown promising results and confirms their potential as a future therapeutic approach in PCa.

### 3.5. Mitochondrial and Signalling Modulators

#### Niclosamide

Niclosamide, initially developed for tapeworm infections, exhibits anticancer potential through multiple mechanisms, including inhibition of mTORC1, STAT3, and Wnt/β-catenin pathways as well as exhibiting antitumour effects by acting as a mitochondrial uncoupler [57]. In PCa, niclosamide indirectly and partially targets AR-V7 and additionally only has a moderate effect on AR-FL [57]. One study has shown it suppresses the FOXM1-mediated DNA damage response, indirectly increasing DNA damage and promoting apoptosis (Figure 2) [56]. Due to poor oral bioavailability, niclosamide was reformulated as niclosamide/PDMX1001. The exact mechanisms of action for niclosamide/PDMX1001 are not fully understood, and the inhibition of AR-V7 by niclosamide is only partly involved in its antitumour effect. In a Phase Ib trial in combination with abiraterone/prednisone involving nine CRPC patients, five achieved ≥50% PSA reductions, and two of the five patients achieved undetectable PSA (<0.01 ng/mL), indicating promising results for niclosamide as a potential CRPC treatment option. Niclosamide/PDMX1001 is currently in Phase II clinical trials, but further studies with larger cohorts are needed to establish safety and efficacy [58].

## 4. Discussion and Future Perspectives

Despite advances in AR-targeted therapies, the emergence of resistance mechanisms, most notably AR gene amplification, point mutations, and the expression of constitutively active splice variants, continues to drive progression to CRPC. This review highlights the breadth of current strategies aimed at overcoming these challenges by targeting multiple domains of the AR or by indirectly modulating AR-dependent transcriptional programmes.

Direct inhibitors targeting the NTD, DBD, and LBD represent complementary approaches that collectively address AR’s structural and functional complexity. NTD inhibitors such as the EPI compounds and QW07 disrupt protein–protein interactions critical for transcriptional activation, including those involving AR variants lacking the LBD. DBD inhibitors, including VPC compounds and PP, further expand the therapeutic scope by preventing AR-chromatin interactions [29, 31]. Meanwhile, LBD-targeting degraders such as PROTACs and CC-94676 provide efficient AR protein elimination, though their dependence on the LBD limits efficacy against splice variants [40, 61].

Emerging indirect strategies, including RIPTACs, BETi like ZEN-3694, and DNA-binding hairpin polyamides, illustrate a paradigm shift from direct AR antagonism toward selective disruption of AR-driven transcriptional networks and epigenetic regulation [46, 52, 55]. These compounds, alongside repurposed agents such as niclosamide, underscore the potential of multi-pathway inhibition to suppress both AR-FL and AR-V signalling. However, these promising approaches also bring key translational challenges, including achieving sufficient nuclear penetration for polyamides, managing toxicity associated with BET inhibition, and overcoming the poor bioavailability seen with compounds like niclosamide [46, 52, 55]. Addressing these limitations will be essential for fully realising the therapeutic potential of indirect AR-pathway modulation.

Moving forward, rational combination therapies that integrate direct and indirect AR-targeting mechanisms may provide the most durable responses. For example, pairing an LBD degrader such as ARV-766 with an NTD inhibitor like EPI-7386 could simultaneously suppress ligand-dependent signalling and the ligand-independent activity of AR-Vs [33, 59]. In this strategy, ARV-766 eliminates LBD-containing isoforms, while EPI-7386 blocks transcriptional output from AR-V7 and other constitutively active variants, preventing escape through LBD-independent pathways.

Combining BET inhibition (e.g., ZEN-3694) with PROTAC-mediated AR degradation offers another complementary approach. BET inhibitors reduce BRD4-driven enhancer activation and AR-dependent transcription, whereas AR degraders remove the receptor itself, enabling deeper pathway suppression, particularly in tumours reliant on enhancer reprogramming and AR-V-driven transcription [33, 71].

Continued optimisation of pharmacokinetics, selectivity, and tumour penetration remains essential for translating these concepts into clinical efficacy.

## 5. Conclusions

Collectively, the diversification of AR-targeting strategies marks significant progress towards overcoming therapeutic resistance in CRPC. Future research should prioritise the mechanistic elucidation of variant-selective inhibition and the clinical evaluation of synergistic combinations. Such efforts will be essential to fully exploit the therapeutic vulnerabilities of AR signalling and improve patient outcomes in advanced PCa.

## References

[1] Prostate Cancer Statistics Cancer Research UK. https://www.cancerresearchuk.org/health-professional/cancer-statistics/statistics-by-cancer-type/prostate-cancer.

[2] James N.D., Tannock I., N’Dow J., Feng F., Gillessen S., Ali S.A., Trujillo B., Al-Lazikani B., Attard G., Bray F. (2024). The Lancet Commission on prostate cancer: Planning for the surge in cases. Lancet.

[3] Brawley S., Mohan R., Nein C.D. (2018). Localized Prostate Cancer: Treatment Options. Am. Fam. Physician.

[4] Nguyen-Nielsen M., Borre M. (2016). Diagnostic and Therapeutic Strategies for Prostate Cancer. Semin. Nucl. Med..

[5] Litwin M.S., Tan H.-J. (2017). The Diagnosis and Treatment of Prostate Cancer: A Review. JAMA.

[6] Perlmutter M.A., Lepor H. (2007). Androgen deprivation therapy in the treatment of advanced prostate cancer. Rev. Urol..

[7] Kunath F., Borgmann H., Blümle A., Keck B., Wullich B., Schmucker C., Sikic D., Roelle C., Schmidt S., Wahba A. (2015). Gonadotropin-releasing hormone antagonists versus standard androgen suppression therapy for advanced prostate cancer A systematic review with meta-analysis. BMJ Open.

[8] Karantanos T., Corn P.G., Thompson T.C. (2013). Prostate cancer progression after androgen deprivation therapy: Mechanisms of castrate-resistance and novel therapeutic approaches. Oncogene.

[9] Chandrasekar T., Yang J.C., Gao A.C., Evans C.P. (2015). Mechanisms of resistance in castration-resistant prostate cancer (CRPC). Transl. Androl. Urol..

[10] Messner E.A., Steele T.M., Tsamouri M.M., Hejazi N., Gao A.C., Mudryj M., Ghosh P.M. (2020). The Androgen Receptor in Prostate Cancer: Effect of Structure, Ligands and Spliced Variants on Therapy. Biomedicines.

[11] Davey R.A., Grossmann M. (2016). Androgen Receptor Structure, Function and Biology: From Bench to Bedside. Clin. Biochem. Rev..

[12] Tan M.E., Li J., Xu H.E., Melcher K., Yong E. (2015). Androgen receptor: Structure, role in prostate cancer and drug discovery. Acta Pharmacol. Sin..

[13] Centenera M.M., Harris J.M., Tilley W.D., Butler L.M. (2008). Minireview: The Contribution of Different Androgen Receptor Domains to Receptor Dimerization and Signaling. Mol. Endocrinol..

[14] Le T.K., Duong Q.H., Baylot V., Fargette C., Baboudjian M., Colleaux L., Taïeb D., Rocchi P. (2023). Castration-Resistant Prostate Cancer: From Uncovered Resistance Mechanisms to Current Treatments. Cancers.

[15] Vellky J.E., Ricke W.A. (2020). Development and prevalence of castration-resistant prostate cancer subtypes. Neoplasia.

[16] Tran C., Ouk S., Clegg N.J., Chen Y., Watson P.A., Arora V., Wongvipat J., Smith-Jones P.M., Yoo D., Kwon A. (2009). Development of a second-generation antiandrogen for treatment of advanced prostate cancer. Science.

[17] Clegg N.J., Wongvipat J., Joseph J., Tran C., Ouk S., Dilhas A., Chen Y., Grillot K., Bischoff E.D., Cai L. (2012). ARN-509: A novel anti-androgen for prostate cancer treatment. Cancer Res..

[18] Maylin Z.R., Nicolescu R.C., Pandha H., Asim M. (2021). Breaking androgen receptor addiction of prostate cancer by targeting different functional domains in the treatment of advanced disease. Transl. Oncol..

[19] Zhu Y., Luo J. (2020). Regulation of androgen receptor variants in prostate cancer. Asian J. Urol..

[20] Antonarakis E.S., Chandhasin C., Osbourne E., Luo J., Sadar M.D., Perabo F. (2016). Targeting the N-Terminal Domain of the Androgen Receptor: A New Approach for the Treatment of Advanced Prostate Cancer. Oncol..

[21] Yang Y.C., Banuelos C.A., Mawji N.R., Wang J., Kato M., Haile S., McEwan I.J., Plymate S., Sadar M.D. (2016). Targeting Androgen Receptor Activation Function-1 with EPI to Overcome Resistance Mechanisms in Castration-Resistant Prostate Cancer. Clin. Cancer Res..

[22] Kyriakopoulos C., Chatta G.S., Laccetti A.L., Iannotti N., Sokolova A., Hotte S.J., Cesano A. (2024). Phase 1/2 trial of oral EPI-7386 (masofaniten) in combination with enzalutamide (Enz) compared to Enz alone in patients with metastatic castration-resistant prostate cancer (mCRPC): Phase 1 (P1) results and phase 2 (P2) design. J. Clin. Oncol..

[23] Hong N.H., Le Moigne R., Banuelos C.A., Mawji N.R., Tam T., Wang J., Andersen R.J., Cesano A., Sadar M.D., Zhou H.-J. (2020). Pre-clinical development of the second-generation N-terminal domain androgen receptor inhibitor, EPI-7386, for the treatment of prostate cancer. Cancer Res..

[24] Obst J.K., Wang J., Jian K., Williams D.E., Tien A.H., Mawji N., Tam T., Yang Y.C., Andersen R.J., Chi K.N. (2019). Revealing Metabolic Liabilities of Ralaniten to Enhance Novel Androgen Receptor Targeted Therapies. ACS Pharmacol. Transl. Sci..

[25] Brand L.J., Olson M.E., Ravindranathan P., Guo H., Kempema A.M., Andrews T.E., Dehm S.M. (2015). EPI-001 is a selective peroxisome proliferator-activated receptor-gamma modulator with inhibitory effects on androgen receptor expression and activity in prostate cancer. Oncotarget.

[26] Peng S., Wang J., Chen H., Hu P., He X.-L., He Y., Wang M., Tang W., He Q., Wang Y.-Y. (2020). Regression of castration-resistant prostate cancer by a novel compound QW07 targeting androgen receptor N-terminal domain. Cell Biol. Toxicol..

[27] Chen Y., Lan T. (2024). N-terminal domain of androgen receptor is a major therapeutic barrier and potential pharmacological target for treating castration resistant prostate cancer: A comprehensive review. Front. Pharmacol..

[28] Sekhon I., Chen G., Piri K., Shinkawa S., Ashong D., Zhang Q., Wang G., Chen Q.-H. (2023). Tricyclic Diterpenoids Selectively Suppress Androgen Receptor-Positive Prostate Cancer Cells. Molecules.

[29] Dalal K., Che M., Que N.S., Sharma A., Yang R., Lallous N., Borgmann H., Ozistanbullu D., Tse R., Ban F. (2017). Bypassing Drug Resistance Mechanisms of Prostate Cancer with Small Molecules that Target Androgen Receptor–Chromatin Interactions. Mol. Cancer Ther..

[30] Radaeva M., Ban F., Zhang F., LeBlanc E., Lallous N., Rennie P.S., Gleave M.E., Cherkasov A. (2021). Development of Novel Inhibitors Targeting the D-Box of the DNA Binding Domain of Androgen Receptor. Int. J. Mol. Sci..

[31] Lim M., Otto-Duessel M., He M., Su L., Nguyen D., Chin E., Alliston T., Jones J.O. (2014). Ligand-independent and tissue-selective androgen receptor inhibition by pyrvinium. ACS Chem. Biol..

[32] Schultz C.W., Nevler A. (2022). Pyrvinium Pamoate: Past, Present, and Future as an Anti-Cancer Drug. Biomedicines.

[33] Chen Q.-H., Munoz E., Ashong D. (2024). Insight into Recent Advances in Degrading Androgen Receptor for Castration-Resistant Prostate Cancer. Cancers.

[34] Zhang Y., Ming A., Wang J., Chen W., Fang Z. (2024). PROTACs targeting androgen receptor signaling: Potential therapeutic agents for castration-resistant prostate cancer. Pharmacol. Res..

[35] Petrylak D.P., McKean M., Lang J.M., Gao X., Dreicer R., Geynisman D.M., Shore15 N.D. (2024). ARV-766, a Proteolysis Targeting Chimera (PROTAC) Androgen Receptor (AR) Degrader, in Metastatic Castration-Resistant Prostate Cancer (mCRPC): Initial Results of a Phase 1/2 Study. J. Clin. Oncol..

[36] Burke M.R., Smith A.R., Zheng G. (2022). Overcoming Cancer Drug Resistance Utilizing PROTAC Technology. Front. Cell Dev. Biol..

[37] Yoshida S., Kajiwara D., Seki M., Tayama M., Tanaka Y., Mizutani H., Fujita R., Yamamura K., Okajima S., Asai M. (2024). TAS3681, an androgen receptor antagonist, prevents drug resistance driven by aberrant androgen receptor signaling in prostate cancer. Mol. Oncol..

[38] De Bono J.S., Cook N., Yu E.Y., Lara P.L.N., Wang J.S., Yamasaki Y., Yamamiya I., Gao P., Calleja E.M., Rathkopf D.E. (2021). First-in-human study of TAS3681, an oral androgen receptor (AR) antagonist with AR and AR splice variant (AR-SV) downregulation activity, in patients (pts) with metastatic castration-resistant prostate cancer (mCRPC) refractory to abiraterone (ABI) and/or enzalutamide (ENZ) and chemotherapy (CT). J. Clin. Oncol..

[39] Nayak S., Norris J.D., Ammirante M., Rychak E., Wardell S.E., Liao D., Toyama B., Kandimalla R., Christoforou A., Tsuji T. (2025). Discovery of BMS-986365, a First-in-Class Dual Androgen Receptor Ligand-Directed Degrader and Antagonist, for the Treatment of Advanced Prostate Cancer. Clin. Cancer Res..

[40] Rathkopf D.E., Patel M.R., Choundhury A.D., Rasco D., Lakhani N., Hawley J.E., Srinivas S., Aparicio A., Narayan V., Runcie K.D. (2025). Safety and clinical activity of BMS-986365 (CC-94676), a dual androgen receptor ligand-directed degrader and antagonist, in heavily pretreated patients with metastatic castration-resistant prostate cancer. Ann. Oncol..

[41] Celgene, A Phase 3, Two-Part, Randomized, Open-label, Adaptive Study Comparing BMS-986365 Versus Investigator’s Choice of Therapy Comprising Either Docetaxel or Second Androgen Receptor Pathway Inhibitor (ARPI), in Participants with Metastatic Castration-Resistant Prostate Cancer (mCRPC)—rechARge. Clinicaltrials.gov, Clinical Trial Registration NCT06764485. Oct. 2025. NCT06764485.

[42] Rathkopf D.E., Patel M.R., Choundhury A.D., Rasco D.W., Lakhani N.J., Hawley J.E., Aparicio A., Narayan V., Srinivas S., Runcie K. (2024). First-in-human phase 1 study of CC-94676, a first-in-class androgen receptor (AR) ligand-directed degrader (LDD), in patients (pts) with metastatic castration-resistant prostate cancer (mCRPC). J. Clin. Oncol..

[43] Ma Z., Zhang C., Shen Q., Zhou J. (2025). RIPTACs for Precision Cancer Therapy: A Novel Modality with the Inspiration of HLD-0915 as the First Candidate in Clinical Trials. J. Med. Chem..

[44] Raina K., Forbes C.D., Stronk R., Rappi J.P., Eastman K.J., Zaware N., Yu X., Li H., Bhardwaj A., Gerritz S.W. (2024). Regulated induced proximity targeting chimeras-RIPTACs-A heterobifunctional small molecule strategy for cancer selective therapies. Cell Chem. Biol..

[45] Therapeutics H. Halda Therapeutics Announces First-in-Human Results for HLD-0915, an Oral RIPTAC^TM^ Therapeutic Demonstrating Encouraging Safety and Anti-Tumor Activity in Metastatic Castration-Resistance Prostate Cancer (mCRPC). Halda Therapeutics. https://haldatx.com/halda-therapeutics-announces-first-in-human-results-for-hld-0915-an-oral-riptac-therapeutic-demonstrating-encouraging-safety-and-anti-tumor-activity-in-metastatic-castration-resistance-prost/.

[46] Ma Z., Bolinger A.A., Zhou J. (2023). RIPTACs: A groundbreaking approach to drug discovery. Drug Discov. Today.

[47] Chou F.-J., Chen Y., Chen D., Niu Y., Li G., Keng P., Yeh S., Chang C. (2019). Preclinical study using androgen receptor (AR) degradation enhancer to increase radiotherapy efficacy via targeting radiation-increased AR to better suppress prostate cancer progression. EBioMedicine.

[48] Hu H., Zhou H., Xu D. (2021). A review of the effects and molecular mechanisms of dimethylcurcumin (ASC-J9) on androgen receptor-related diseases. Chem. Biol. Drug Des..

[49] Yamashita S., Lai K.-P., Chuang K.-L., Xu D., Miyamoto H., Tochigi T., Pang S.-T., Li L., Arai Y., Kung H.-J. (2012). ASC-J9 Suppresses Castration-Resistant Prostate Cancer Growth through Degradation of Full-length and Splice Variant Androgen Receptors. Neoplasia.

[50] Arora A., Kumar S., Kumar S., Kumar R., Prasad A.-K. (2022). Chemical Features and Therapeutic Applications of Curcumin (A Review). Russ. J. Gen. Chem..

[51] Aggarwal R.R., Schweizer M.T., Nanus D.M., Pantuck A.J., Heath E.I., Campeau E., Attwell S., Norek K., Snyder M., Bauman L. (2020). A Phase Ib/IIa Study of the Pan-BET Inhibitor ZEN-3694 in Combination with Enzalutamide in Patients with Metastatic Castration-resistant Prostate Cancer. Clin. Cancer Res..

[52] Attwel S., Jahagirdar R., Norek K., Calosing C., Tsujikawa L., Kharenko O.A., Patel R.G., Gesner E.M., Corey E., Nguyen H.M. (2016). Abstract LB-207: Preclinical characterization of ZEN-3694, a novel BET bromodomain inhibitor entering phase I studies for metastatic castration-resistant prostate cancer (mCRPC). Cancer Res..

[53] Yang F., Nickols N.G., Li B.C., Marinov G.K., Said J.W., Dervan P.B. (2013). Antitumor activity of a pyrrole-imidazole polyamide. Proc. Natl. Acad. Sci. USA.

[54] Kurmis A.A., Yang F., Welch T.R., Nickols N.G., Dervan P.B. (2017). A pyrrole-imidazole polyamide is active against enzalutamide-resistant prostate cancer. Cancer Res..

[55] Yang F., Nickols N.G., Li B.C., Szablowski J.O., Hamilton S.R., Meier J.L., Dervan P.B. (2013). Animal Toxicity of Hairpin Pyrrole-Imidazole Polyamides Varies with the Turn Unit. J. Med. Chem..

[56] Kim M.Y., Jung A.R., Shin D., Kwon H., Cho H.J., Ha U.-S., Hong S.-H., Lee J.Y., Kim S.W., Park Y.H. (2021). Niclosamide exerts anticancer effects through inhibition of the FOXM1-mediated DNA damage response in prostate cancer. Am. J. Cancer Res..

[57] Sakellakis M. (2023). Niclosamide in prostate cancer: An inhibitor of AR-V7, a mitochondrial uncoupler, or more?. Cancer Treat. Res. Commun..

[58] Parikh M., Liu C., Wu C.-Y., Evans C.P., Dall’Era M., Robles D., Lara P.N., Agarwal N., Gao A.C., Pan C.-X. (2021). Phase Ib trial of reformulated niclosamide with abiraterone/prednisone in men with castration-resistant prostate cancer. Sci. Rep..

[59] Siu L.L., Rasco D.W., Vinay S.P., Romano P.M., Menis J., Opdam F.L., Heinhuis K.M., Egger J.L., Gorman S.A., Parasrampuria R. (2019). 438O—METEOR-1: A phase I study of GSK3326595, a first-in-class protein arginine methyltransferase 5 (PRMT5) inhibitor, in advanced solid tumours. Ann. Oncol..

[60] Nicolescu R.C.B., Maylin Z.R., Pérez-Areales F.J., Iegre J., Pandha H.S., Asim M., Spring D.R. (2023). Hybrid Androgen Receptor Inhibitors Outperform Enzalutamide and EPI-001 in in vitro Models of Prostate Cancer Drug Resistance. Chemmedchem.

[61] Scher H.I., Fizazi K., Saad F., Taplin M.-E., Sternberg C.N., Miller K., de Wit R., Mulders P., Chi K.N., Shore N.D. (2012). Increased survival with enzalutamide in prostate cancer after chemotherapy. N. Engl. J. Med..

[62] Asim M., Massie C.E., Orafidiya F., Pértega-Gomes N., Warren A.Y., Esmaeili M., Selth L.A., Zecchini H.I., Luko K., Qureshi A. (2015). Choline Kinase Alpha as an Androgen Receptor Chaperone and Prostate Cancer Therapeutic Target. J Natl Cancer Inst..

[63] Clinical Trial: NCT02566772—My Cancer Genome. NCT02566772.

[64] Wang Z.-Q., Zhang Z.-C., Wu Y.-Y., Pi Y.-N., Lou S.H., Liu T.-B., Lou G., Yang C. (2023). Bromodomain and extraterminal (BET) proteins: Biological functions, diseases and targeted therapy. Signal Transduct. Target. Ther..

[65] Jia X., Han X. (2023). Targeting androgen receptor degradation with PROTACs from bench to bedside. Biomed. Pharmacother..

[66] Li Y., Chu Y., Shi G., Wang X., Ye W., Shan C., Wang D., Zhang D., He W., Jiang J. (2022). A novel inhibitor of ARfl and ARv7 induces protein degradation to overcome enzalutamide resistance in advanced prostate cancer. Acta Pharm. Sin. B.

[67] Watson P.A., Chen Y.F., Balbas M.D., Wongvipat J., Socci N.D., Viale A., Kim K., Sawyers C.L. (2010). Constitutively active androgen receptor splice variants expressed in castration-resistant prostate cancer require full-length androgen receptor. Proc. Natl. Acad. Sci. USA.

[68] Lai K.-P., Huang C.-K., Chang Y.-J., Chung C.-Y., Yamashita S., Li L., Lee S.-O., Yeh S., Chang C. (2013). New Therapeutic Approach to Suppress Castration-Resistant Prostate Cancer Using ASC-J9 via Targeting Androgen Receptor in Selective Prostate Cells. Am. J. Pathol..

[69] Tian H., Chou F.-J., Tian J., Zhang Y., You B., Huang C.-P., Yeh S., Niu Y., Chang C. (2021). ASC-J9^®^ suppresses prostate cancer cell proliferation and invasion via altering the ATF3-PTK2 signaling. J. Exp. Clin. Cancer Res. CR.

[70] Ali H.A., Li Y., Bilal A.H.M., Qin T., Yuan Z., Zhao W. (2022). A Comprehensive Review of BET Protein Biochemistry, Physiology, and Pathological Roles. Front. Pharmacol..

[71] Zen-3694—My Cancer Genome. https://www.mycancergenome.org/content/drugs/zen-3694/.

